# Evaluation of *In Vivo* Wound Healing Activity of *Bacopa monniera* on Different Wound Model in Rats

**DOI:** 10.1155/2013/972028

**Published:** 2013-07-29

**Authors:** S. Murthy, M. K. Gautam, Shalini Goel, V. Purohit, H. Sharma, R. K. Goel

**Affiliations:** ^1^Department of Pharmacology, Institute of Medical Sciences, Banaras Hindu University, Varanasi 221005, India; ^2^Department of Pathology and Lab Medicine, Medanta-The Medicity, Sector 38, Gurgaon 122001, India

## Abstract

Wound healing effects of 50% ethanol extract of dried whole plant of *Bacopa monniera* (BME) was studied on wound models in rats. BME (25 mg/kg) was administered orally, once daily for 10 days (incision and dead space wound models) or for 21 days or more (excision wound model) in rats. BME was studied for its *in vitro* antimicrobial and *in vivo* wound breaking strength, WBS (incision model), rate of contraction, period of epithelization, histology of skin (excision model), granulation tissue free radicals (nitric oxide and lipid peroxidation), antioxidants (catalase, superoxide dismutase, and reduced glutathione), acute inflammatory marker (myeloperoxidase), connective tissue markers (hydroxyproline, hexosamine, and hexuronic acid), and deep connective tissue histology (dead space wound). BME showed antimicrobial activity against skin pathogens, enhanced WBS, rate of contraction, skin collagen tissue formation, and early epithelization period with low scar area indicating enhanced healing. Healing effect was further substantiated by decreased free radicals and myeloperoxidase and enhanced antioxidants and connective tissue markers with histological evidence of more collagen formation in skin and deeper connective tissues. BME decreased myeloperoxidase and free radical generated tissue damage, promoting antioxidant status, faster collagen deposition, other connective tissue constituent formation, and antibacterial activity.

## 1. Introduction

Wounds are physical injuries that result in an opening or breaking of the skin. Proper healing of wounds is essential for the restoration of disrupted anatomical stability and disturbed functional status of the skin. Repair of injured tissues occurs as a sequence of events, which includes inflammation, proliferation, and migration of different cell types [1]. The inflammation stage begins immediately after injury, first with vasoconstriction that favors homeostasis and releases inflammation mediators. The proliferative phase is characterized by granulation tissue proliferation formed mainly by fibroblast and the angiogenesis process. The remodeling stage is characterized by reformulations and improvement in the components of the collagen fibre that increases the tensile strength [2]. Factors that contribute to causation and perpetuation of the chronicity of wounds include repeated trauma, poor perfusion or oxygenation, and excessive inflammation [3]. Imbalance in free radical generations and antioxidants has been observed to induce oxidative stress and tissue damage and delayed wound healing. Therefore, elimination of ROS could be an important strategy in healing chronic wounds [4].

*Bacopa monniera* (BM, Scrophulariaceae) called as water hyssop is a prostrate herb, commonly found in wet or marshy habitats and along the stream and river margins throughout India. The Charaka Samhita considers BM (Synonyms: *Herpestis monniera*) as medhya rasayana and Ayurvedic texts advocate the use of BM in ascites, enlarged spleen, indigestion, inflammation and leprosy, and so forth and, as a result, researchers have evaluated its sedative and tranquillizing, cognition, antidepressant and antianxiety, antiepileptic, antioxidant and adaptogenic, antiulcer, and anti-*Helicobacter *properties [5–13]. Recently, alcoholic extract of *Bacopa monniera* and its isolated constituent Bacoside-A were screened for wound healing activity by excision, incision, and dead space wound on Swiss albino rats and were found to enhance wound healing in terms of increase in tensile strength, wound epithelization, and connective tissue formation [14].

The present study was, therefore, undertaken to do an in-depth study on the wound healing activities of 50% ethanol extract of whole plant of *Bacopa monniera *in incision, excision, and dead space wound models in rats when given by oral route.

## 2. Materials and Methods

### 2.1. Animals

Inbred Charles-Foster albino rats (160–180 g) of either sex were obtained from the central animal house of Institute of Medical Sciences, Banaras Hindu University, Varanasi. They were kept in the departmental animal house at 26 ± 20°C and relative humidity 44–56%, light and dark cycles of 10 and 14 h, respectively, for one week before and during the experiments. Animals were provided with standard rodent pellet diet (Pashu Aahar Vihar, Ramnagar, Varanasi) and water *ad libitum*. “Principles of laboratory animal care” (NIH publication no. 82-23, revised 1985) guidelines were followed. Approval from the Institutional Animal Ethical Committee was taken prior to the experimental work (Notification no. Dean/2010-11/275 dated 13.10.2010). 

### 2.2. Plant Material and Preparation of Extract

The whole plant of *Bacopa monniera *(BM) (Ayurvedic Gardens, Banaras Hindu University) was collected during April–June and identified with the standard sample preserved in the department of Dravyaguna, Institute of Medical Sciences, Banaras Hindu University, Varanasi. 50% ethanolic extract of BM (BME) was prepared by adding 500 g of dried, crushed, and powdered whole plant of BM in 1000 mL of 50% ethanol in a round bottom flask and was kept at room temperature for 3 days in shade. The extract was filtered and the previous procedure was repeated twice. The extract filtrate so obtained was pooled and evaporated on water bath till it dried. The yield of BME was about 28.16% (w/w).

### 2.3. Drug and Chemicals

Vitamin E (Merck Ltd., Mumbai, India) and all the other chemicals and reagents were used of analytical grade.

### 2.4. Dose Selection and Treatment Protocol

A preliminary dose-response effect using BME was first undertaken to study the wound breaking strength, in incision wound model, in rat. Graded doses of BME 12.5, 25, and 50 mg/kg were administered once daily orally for 10 days in rats following induction of incision wound. The sutures were removed on 7th day of experiment and wound breaking strength (WBS) was measured on 10th postwounding day. The result of the dose response study in incision wound model indicated that 25 mg/kg of BME had the optimal effect. Therefore, dose of 25 mg/kg of BME was chosen for further study on various physical, biochemical, and histopathological parameters of wound healing in rat dead space wound models. BME (25 mg/kg) and the standard drug Vitamin E (VTE; 200 mg/kg), suspended in 0.5% carboxy methyl cellulose (CMC) in distilled water, were given orally once daily from day 1, 4 hours after the induction of excision and dead space wounds. The animals received CMC/BME/VTE orally with the help of an orogastric tube in the volume of 1 mL/100 g body weight for 10 days for dead space wound and incision wound studies and 20 days or till the period of complete epithelization for excision wound study.

### 2.5. Wound Healing Studies

#### 2.5.1. Incision Wound Model

Two parallel six cm paravertebral incisions were made through the full thickness of the skin, 1 cm lateral to the midline of vertebral column after giving anaesthesia [15]. Wounds were closed with interrupted sutures, 1 cm apart, with surgical suture. The sutures were removed on the 7th postwounding day. Wound breaking strength (WBS) was measured on the 10th postwounding day in anaesthetized rats. A line was drawn on either side of the incision line 3 mm away from the wound. Two Allis forceps were firmly applied on to the line facing each other. One of the forceps was fixed, while the other was connected to a freely suspended lightweight polypropylene graduated container through a string run over to a pulley. Standard weights were put slowly and steadily into the container. A gradual increase in weight was transmitted to the wound site pulling apart the wound edges. As and when the wound just opened up, the weight was stopped and noted.

#### 2.5.2. Excision Wound Model

Rats were anesthetized with ketamine (30 mg/kg, ip) and an area of about *≈*500 mm^2^ was marked on the back of the rat by a standard ring. Full thickness of the marked skin was then cut carefully. Wounds were traced on 1 mm^2^ graph paper on the day of wounding and subsequently at a gap period of 4 days till 12th day, then on the alternate days until healing was complete. Changes in wound area were measured regularly and the rate of wound contraction calculated as given in the formula below. Significance in wound healing of the test groups is derived by comparing healed wound area on respective days with healed wound area of control group. The period of epithelization, that is, day of fall of eschar and the scar area, was also noted down [15]:

(1) %  wound  contraction=[Healed  area°Total  wound  area  ]×100,(Healed  area°  =original  wound  area− present  wound  area).

#### 2.5.3. Dead Space Wound Model

Rats were anesthetized with ketamine and 1 cm incision was made on dorsolumbar part of the back. Two polypropylene tubes (0.5 × 2.5 cm^2^ each) were placed in the dead space of lumbar region of rat on each side, and wounds were closed with a suture material. On the 10th postwounding day, the animals were sacrificed and granulation tissue formed on and around the implanted tubes was carefully dissected out, weighed, and processed for the estimation of free radicals, antioxidants, and collagen tissue parameters [15].

### 2.6. Estimation of Granulation Tissue Free Radical and Antioxidant

Antioxidants—superoxide dismutase, SOD [16]; catalase, CAT [17] and reduced glutathione, GSH [18]; free radicals—lipid peroxidation, LPO [19] and nitric oxide, NO [20] and acute inflammatory marker, myeloperoxidase (MPO) [21], and protein [22] were estimated in wet granulation tissue homogenates. Briefly, the wet granulation tissues were homogenized in a glass Teflon homogenizer (10% w/v) at 4°C in Phosphate buffered saline (PBS, pH 7) used for the estimation of protein, free radicals, and antioxidants. The assay of SOD is based on the inhibition of the formation of NADH-phenazine methosulphate-nitro blue tetrazolium formazan. One unit of enzyme activity is defined as the amount of enzyme that gave 50% inhibition of nitro blue tetrazolium reduction in one minute. CAT measurement was done based on the ability of catalase to oxidize hydrogen peroxide. One unit (U) of catalase is the enzyme, which decomposes one mM of H_2_O_2_/min at 25°C. GSH activity in the homogenate was estimated by the ability of GSH to reduce DTNB within 5 min of its addition against blank. LPO levels were estimated in terms of malondialdehyde (MDA) released during lipid peroxidation Nitrites and nitrates are formed as end products of reactive nitrogen products during NO formation which are measured by using Griess reagent. 

For myeloperoxidase (MPO) estimation, granulation tissue (5% w/v) was homogenized in 0.5% hexadecyltrimethylammonium bromide (HTAB, Sigma-Aldrich, Co., St. Louis, MO, USA) with 50 mM potassium phosphate buffer (pH 6). The previous homogenate was freeze-thawed three times, sonicated for 10 seconds, and then centrifuged at 14000 ×g for 45 minutes at 4°C and the resulting supernatant was used for estimation of MPO. A unit of MPO activity is defined as that converting 1 *μ*mol of H_2_O_2_ to water in 1 min at 25°C.

### 2.7. Estimation of Connective Tissue Parameters

Approximately 250 mg of wet tissue was dried at 50°C for 24 h. It was weighed and kept in glass stoppered test tubes. To each tube containing 40 mg of the dried granulation tissue, 1 mL of 6 N HCl was added. The tubes were then kept on boiling water bath for 24 h (12 h each day for two days) for hydrolysis. The hydrolysate was then cooled and excess of acid was neutralized by 10 N NaOH using phenolphthalein as indicator. The volume of neutral hydrolysate was diluted to a concentration of 20 mg/mL with distilled water. The final hydrolysate was used for the estimation of hydroxyproline, hexosamine, and hexuronic acid following the standard curve prepared using the proper substrate.

#### 2.7.1. Hydroxyproline (HPR)

To the each tube, 0.3 mL each of hydrolysate, 2.5 N NaOH, 0.01 M CuSO_4_, and 6% H_2_O_2_ were added. Tubes were shaken vigorously and placed immediately in water bath at 80°C. After 15 minutes, tubes were removed and cooled for 5 minutes in cold water. 0.6 mL of freshly prepared 5% solution of paradimethyl amino-benzaldehyde in n-Propanol and 1.2 mL of 3 N H_2_SO_4_ was added. The test tubes were once again placed in a hot water bath at 75°C for 15 minutes and then cooled for 5 minutes under running stream of water. Color intensity was measured at 540 nm against the blank. Hydroxyproline content in the tissue was estimated as per standard curve prepared with standard 4-Hydroxy-L-proline (HiMedia Laboratories Pvt. Ltd., Mumbai, India), from 75 to 900 *μ*g/0.3 mL using 3 mg/mL working solution [23].

#### 2.7.2. Hexosamine (HXA)

0.05 mL of hydrolyzed fraction was diluted to 0.5 mL with distilled water. To this was added 0.5 mL of acetyl acetone reagent and heated in boiling water bath for 20 min then cooled under tap water. To this 1.5 mL of 95% alcohol was added, followed by an addition of 0.5 mL of Ehrlich's reagent. The reaction was allowed for 30 minutes to complete. Color intensity was measured at 530 nm against the blank. Hexosamine content of the samples was determined from the standard curve prepared with D (+) glucosamine hydrochloride (HiMedia Laboratories Pvt. Ltd., Mumbai, India), from 5 to 50 *μ*g/0.5 mL using 100 *μ*g/mL working solution [24]. 

#### 2.7.3. Hexuronic Acid (HUA)

2.5 mL of 0.025 M Borax in concentrated sulphuric acid is placed in stoppered tubes fixed in a rack and cooled to 4°C. 0.125 mL of hydrolysate was diluted 0.5 mL by adding distilled water. Now, this 0.5 mL of hydrolysate is layered carefully on Borax-sulphuric acid mixture kept in rack at 4°C. The tubes were closed with glass stoppers and then shaken, first slowly then vigorously, with constant cooling by placing tubes in ice container. The tubes were then heated for 10 min in a vigorously boiling water bath and cooled to room temperature. Thereafter, 0.1 mL of 0.125% carbazole reagent in absolute alcohol was then added to each tube, shaken, again heated in the boiling water bath for further 15 min, and then cooled to room temperature. Color intensity was measured at 530 nm against the blank. Hexuronic acid content of the samples was determined from the standard curve prepared with D (+) Glucurono-6, 3-lactone (HiMedia Laboratories Pvt. Ltd., Mumbai, India), from 5 to 40 *μ*g/0.5 mL using 100 *μ*g/mL working solution [25].

### 2.8. Histopathology

The cross-sectional full-thickness skin specimens and deep granulation tissues from the implanted tube were collected on the 10th day of the experiment for the histopathological alterations. Samples were fixed in 10% buffered formalin, processed, blocked with paraffin, then sectioned into 5 *μ*m sections, and stained with hematoxylin and eosin.

### 2.9. Antimicrobial Susceptibility and Minimum Inhibitory Concentration (MIC)

*In vitro* antibacterial susceptibility test of BME was done using serial concentrations of 50, 100, 150, and 200 mg/mL following the approved standards of the National Committee for Clinical Laboratory Standards (NCCL) [26] against common skin bacteria *Staphylococcus aureus* (ATCC 25323), *Staphylococcus epidermidis*, and *Pseudomonas aeruginosa* obtained from the American Type Culture Collection (ATCC) and clinical strain preserved at Department of Microbiology, Institute of Medical Sciences, BHU, Varanasi, India, following the disk diffusion method [27] while minimum inhibitory concentration (MIC) was performed by microdilution method [28]. Briefly, 24 h old culture of selected microbes was adjusted to 0.5 McFarland standard in sterile normal saline to achieve concentration of ~10^7^ (colony forming units) CFU/mL. Standard antibiotics used as positive control. Dimethylsulfoxide (DMSO) was used as negative control. MIC was determined by microbroth dilution method. Specifically 0.1 mL of standardized inoculums of bacteria (1-2 × 10^7^ CFU/mL) was added in each well of microtiter plate which was incubated aerobically at 37°C for bacterial growth for 18–24 h. The lowest concentration (highest dilution) of the extract that produced no visible bacterial growth (no turbidity) when compared with the control was regarded as MIC. 

### 2.10. Statistical Analysis

Statistical comparison was performed using either unpaired *t-*test or one-way analysis of variance (ANOVA) followed by Dunnett's test for multiple comparisons" instead of "for multiple comparisons verses control group was done by Dunnett's test. All statistical analysis was performed using SPSS statistical version 16.0 software package (SPSS Inc., USA). 

## 3. Results

### 3.1. Incision Wound Model

Control rats showed WBS as 283.3 ± 18.6 g on 10th postwound day. BME 12.5, 25, 50 mg/kg treated rats showed WBS as 353.3 ± 19.3 g, 373.3 ± 13.8 g, 375.0 ± 14.8 g (*P* < 0.05 to *P* < 0.01), respectively, while vitamin E (VTE, 200 mg/kg) treated rats showed WBS as 405.0 ± 21.1 g (*P* < 0.01).

### 3.2. Excision Wound Model

**Figure 1:**
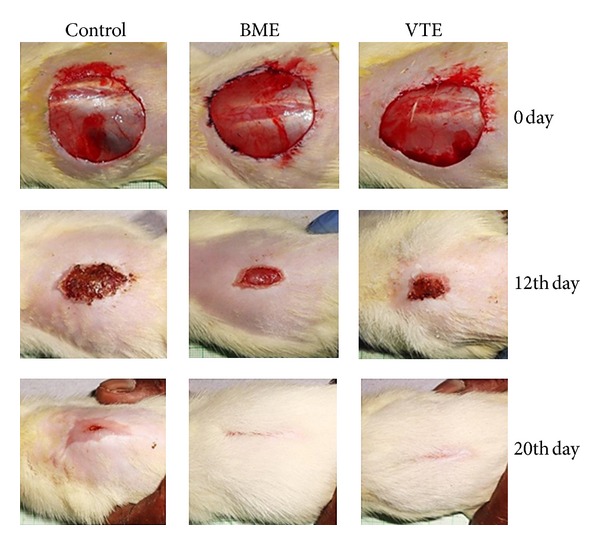
Photographic representation of contraction rate showing percent wound contraction area on different postexcision days of control, BME (25 mg/kg), and VTE (200 mg/kg) treated rats.

Rate of wound contraction in control rats was 21.6% to 68.3% from day 4 to day 12 and 80.6% to 98.1% from day 14 to day 20, while complete epithelization and healing were observed on day 24. The average number of days that took for the shedding of eschar without leaving any residual raw wound in these rats was 12.7 days and mean of scar area after completing healing was 99.8 mm^2^. The percent rate of wound contraction in rats, treated orally with BME (25 mg/kg), was from 32.2% on day 4 to 85.4% on day 12 and 92.1% to 100% from day 14 to day 20, respectively, while VTE treated rats showed increase in wound contraction from 32.4% on day 4 to 87.6% on day 12 and 92.2% to 100% from day 14 to day 20, respectively. The mean epithelization period and scar area observed with BME were 10.3 days and 75.2 mm^2^ while the mean epithelization period and scar area observed with VTE were 10 days and 74.3 mm^2^, respectively. BME treated rats, thus, showed faster healing which was comparable with VTE treated group (Table 1 & Figure 1).

**Figure 2:**
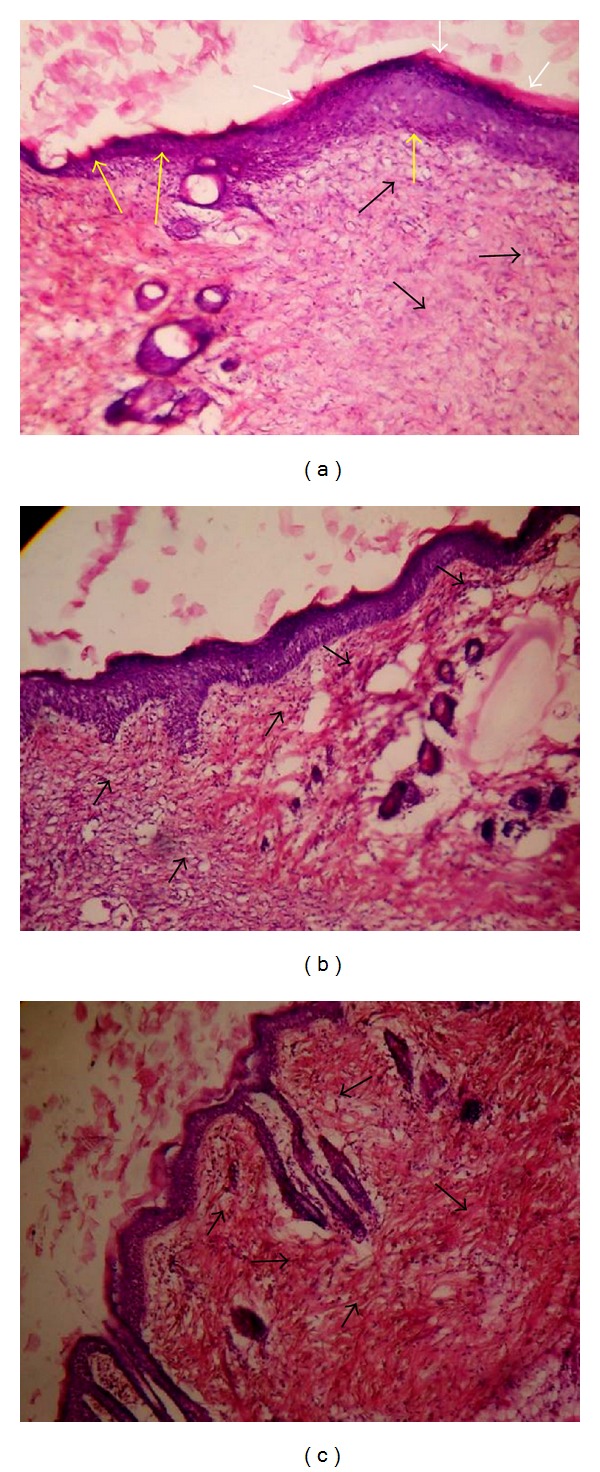
Histopathology of skin at day 10 stained with H&E (100x). (a) Skin of control rat showing ulceration and edema showed by white arrow, early epithelization showed by yellow arrow, and granulation tissue and abundance of mononuclear inflammatory cells showed by black arrow. (b) BME treated rats showing large amount of granulation tissue by black arrow, small number of mononuclear inflammatory cells, and restoration of adnexa and extensive fibrosis. (c) VTE treated rats showing healed skin structures with well-formed, near to normal epidermis, restoration of adnexa, and extensive fibrosis and collagen tissue within the dermis.

Histology of excision biopsy of skin wound at day 10 showed healed skin structures with normal epithelization, restoration of adnexa and fibrosis within the dermis in BME and VTE treated groups while the control group lag behind treated group in formation of the amount of ground substance in the granulation tissue, as observed in tissue sections (Figure 2).

### 3.3. Dead Space Wound Model

BME caused an increase in wet weight mg per 100 g body weight and protein mg/g granulation tissue by 17.6% (*P* < 0.05) and 18.7% (*P* < 0.05), respectively, compared with control group. BME effect was comparable with that of VTE on the previous parameters (Table 2).

### 3.4. Wet Granulation Tissue Antioxidants, Free Radicals, and Myeloperoxidase

BME showed significant increase in the level of antioxidants, GSH, SOD, and CAT while free radicals, LPO and NO, and acute inflammatory marker, MPO were decreased. The results with BME were comparable with VTE on the previous soft-tissue parameters (Table 2). 

### 3.5. Dry Connective Tissue HPR, HXA, and HUA

Dry weight of granulation tissue and protein content were increased by 19.4% and 18.1% (*P* < 0.05), respectively, in BME treated groups compared with control group. HPR, HXA, and HUA were significantly increased in BME treated group by 59.8%, 59.1%, and 153%, respectively, compared to control group. The results with BME were comparable with VTE on the previous connective tissue parameters (Table 3).

**Figure 3:**
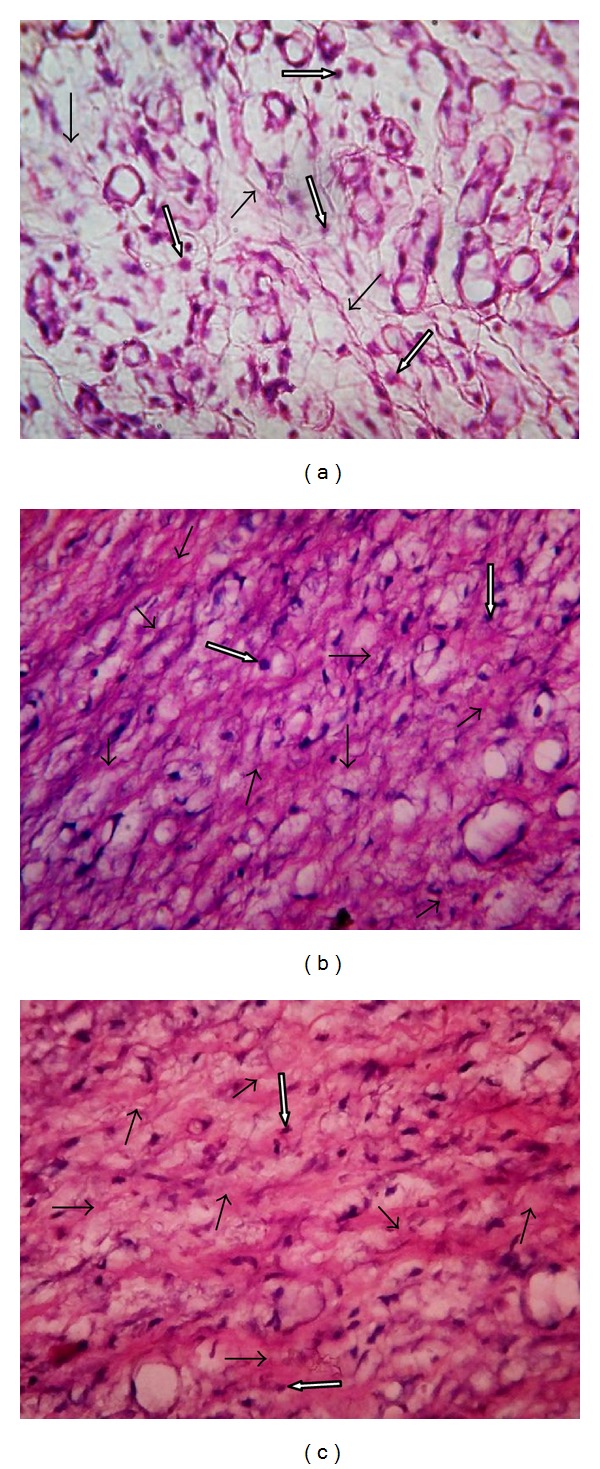
Histopathology of granulation tissue at day 10 stained with H&E (100x). (a) Granulation tissue of control rat showed mononuclear inflammatory cells by white arrow, scattered abundance of eosinophilic fibroblasts showed by black arrow. (b) BME treated showing large number of collagen tissue (fibrosis) and neovascularisation with minimal inflammatory cells. (c) VTE treated showing near to normal features, collagen tissue (fibrosis), and neovascularisation.

Histology of granulation tissue of deeper structure of control rat showed mononuclear inflammatory cells, scattered fibroblasts (minimal fibrosis), and few proliferating vasculature in granulation tissue, while the granulation tissue of rats treated with BME and VTE showed abundance of eosinophilic collagen tissue and neovascularisation with few inflammatory cells indicative of healing by fibrosis (Figure 3).

### 3.6. Antimicrobial Susceptibility and MIC

BME showed positive susceptibility test against common skin bacteria *Staphylococcus aureus* (ATCC 25323), *Staphylococcus epidermidis*, and *Pseudomonas aeruginosa*, showing zone of inhibition ≥10 mm, at 200 mg/mL. BME had least minimum inhibitory concentration (MIC) of 0.39 mg/mL against *Staphylococcus aureus* (ATCC 25323) and 3.125 mg/mL against *Pseudomonas aeruginosa*, whereas MIC of BME against other organisms ranged from 6.25 to 25.0 mg/mL. 

## 4. Discussion

Wound represents a major health problem, both in terms of morbidity and mortality. The processes involved in wound healing are epithelization, contraction, and connective tissue deposition. The healing process depends, to a large extent, on the regulated biosynthesis and deposition of new collagens and their subsequent maturation [15]. In the tissue repair process, inflammatory cells promote the migration and proliferation of endothelial cells, leading to neovascularisation of connective tissue cells which synthesize extracellular matrices including collagen, and of keratinocytes resulting to reepithelialization of the wounded tissue [29]. Inflammation, collagen maturation, and scar formation are some of the many phases of wound healing, which run concurrently but independent of each other. 

In incision wound study, BME showed an increase in breaking strength which may be due to the increase in collagen concentration and stabilization of the fibres [30]. The collagen molecules synthesized are laid down at the wound site and become cross-linked to form fibres. Wound strength is acquired from both remodeling of collagen and the formation of stable intra- and intermolecular crosslinks. BME showed greater breaking strength which may be due to increased collagen synthesis as found in the dead space wound study.

In excision wound, BME showed faster healing compared with control group. Further, excision biopsy of skin wound at day 10 showed healed skin structures with normal epithelization, restoration of adnexa and fibrosis within the dermis in BME and VTE treated groups, while the control group lags behind treated group in formation of the amount of ground substance in the granulation tissue. The faster wound contraction by BME may be due to stimulation of interleukin-8, an inflammatory *α*-chemokine which affects the function and recruitment of various inflammatory cells, fibroblasts and keratinocytes, and may increase the gap junctional intracellular communication in fibroblasts, and induces a more rapid maturation of granulation tissue [31]. 

Collagen is the predominant extracellular protein in the granulation tissue of a healing wound and there is a rapid increase in the synthesis of this protein in the wound area soon after an injury. Breakdown of collagen liberates free hydroxyproline and its peptides. Measurement of this hydroxyproline, therefore, has been used as an index of collagen turnover. The biochemical data of dead space wound study showed an increase in wet tissue weight and protein per g tissue in BME treated groups. The increased hydroxyproline content in the dead space wounds has indicated faster collagen turnover leading to rapid healing with concurrent increase in the breaking strength of the treated wounds. Hexosamine and hexuronic acids are matrix molecules, which act as ground substratum for the synthesis of new extracellular matrix. The glycosaminoglycans are known to stabilize the collagen fibres by enhancing electrostatic and ionic interactions with it and possibly control their ultimate alignment and characteristic size. Their ability to bind and alter protein-protein interactions has identified them as important determinants of cellular responsiveness in development, homeostasis, and disease [32]. In our study, hexuronic acid and hexosamine concentrations which are the components of glycosaminoglycans were significantly increased with BME when compared with control indicating stabilization of collagen fibres. 

Experimental and clinical lines of evidence suggest that chronic wound undergoes substantial oxidative stress by neutrophils-derived oxidants and MPO activity, both of which contribute markedly to tissue damage during chronic wound inflammation [33]. Over production of reactive oxygen species (ROS) results in oxidative stress thereby causing cytotoxicity and delayed wound healing and elimination of ROS could be an important strategy in healing of chronic wounds [4]. Therefore, estimation of antioxidants like GSH, SOD, and CAT in granulation tissues is relevant because the antioxidants have been reported to hasten wound healing by decreasing the free radicals [34]. Our studies on the antioxidants, free radicals, and MPO status revealed that BME had significant antioxidant activity, reduced MPO and free radicals stress, helped to prevent inflammation and oxidative damage, and promoted the healing process. 

The preliminary phytochemical analysis of *Bacopa monniera* (BM) revealed the presence of saponins, glycosides, and alkaloids [35]. Animal research has shown that the phytoconstituents present in BM extract modulate the expression of certain enzymes involved in generation and scavenging of reactive oxygen species in the brain [7]. BM's antioxidant properties and its ability to balance superoxide dismutase and catalase levels were postulated to account for this effect [8]. BM by modulating the extent of lipid peroxidation and enhancing the antioxidant status has DNA protective effects; hence, it is believed to prevent cell damage [36]. Extracts of BM have been reported to have a broad spectrum of antibacterial activity [37], which seemed to have beneficial effects on wound healing. BME was found to show antibacterial activity against *Staphylococcus aureus, Staphylococcus epidermidis*,and* Pseudomonas aeruginosa *and least MIC was found with BME against *Staphylococcus aureus*. 

## 5. Conclusion

Thus, in our present study involving three different wound models, which included observation of different physical, histological, biochemical parameters, and antimicrobial activity, indicated the wound healing activity in the 50% ethanolic extract of dried whole plant of *Bacopa monniera*. The healing effects seemed to be due to decreased free radical generated tissue damage, promoting effects on antioxidant status, faster collagen deposition, and other connective tissue constituent formation, and antibacterial activity.

## Figures and Tables

**Table 1:** Effect of BME and VTE on wound contraction, epithelization period, and scar area in excision wound.

Oral treatment (mg/kg, od)	Wound area in mm^2^/rat (% contraction)									Epithelization period (days)	Scar area (mm^2^)
	0 day	4th day	8th day	12th day	14th day	16th day	18th day	20th day	22nd day		
Control 0.5% CMC	532.8 ± 6.93 (0.00)	417.7 ± 6.77 (21.6 ± 1.0)	322.3 ± 10.8 (39.5 ± 1.73)	168.5 ± 10.1 (68.3 ± 2.03)	103.3 ± 5.79 (80.6 ± 1.16)	81.2 ± 2.36 (84.8 ± 0.48)	36.2 ± 1.25 (93.2 ± 0.20)	10.3 ± 0.67 (98.1 ± 0.15)	3.17 ± 0.75 (99.4 ± 0.15)	12.7 ± 0.67	99.8 ± 4.92
BME 25	530.3 ± 7.15 (0.00)	359.2 ± 12.2^b^ (32.2 ± 2.71)	217.8 ± 20.8^b^ (59.0 ± 3.96)	77.2 ± 5.21^c^ (85.4 ± 1.08)	42.0 ± 3.86^c^ (92.1 ± 0.72)	22.3 ± 2.33^c^ (95.8 ± 0.46)	4.60 ± 0.56^c^ (99.0 ± 0.09)	0.0 ± 0.0^c^ (100.0)	0.0 ± 0.0^c^ (100.0)	10.3 ± 0.49^a^	75.2 ± 4.04^b^
VTE 200	550.0 ± 7.02 (0.00)	371.7 ± 12.1^b^ (32.4 ± 2.03)	229.8 ± 14.2^c^ (58.3 ± 2.48)	68.3 ± 2.55^c^ (87.6 ± 0.49)	42.8 ± 6.13^c^ (92.2 ± 1.12)	19.7 ± 3.02^c^ (96.4 ± 0.55)	3.83 ± 0.98^c^ (99.3 ± 0.17)	0.0 ± 0.0^c^ (100.0)	0.0 ± 0.0^c^ (100.0)	10.0 ± 0.58^a^	74.3 ± 3.98^b^

Values are mean ± SEM (Percent) of 6 rats in each group. ^a^ *P* < 0.05, ^b^ *P* < 0.01, and ^c^ *P* < 0.001 compared to respective day control group (statistical analysis was done by one-way analysis of variance followed by Dunnett's test for multiple comparisons).

**Table 2:** Effect of BME and VTE on wet granulation tissue weight, protein, free radicals (LPO and NO), antioxidants (GSH, SOD, and CAT), and myeloperoxidase (MPO).

Oral treatment (mg/kg, od × 10 day)	Wet tissue mg/100 g bw	Protein mg/g tissue	Antioxidants			Free radicals		Myeloperoxidase
			GSH nM/mg protein	SOD IU/mg protein	CAT mU/mg protein	LPO nM/mg protein	NO nM/mg protein	MPO mU/mg protein
Control 0.5% CMC	359.3 ± 18.3	48.6 ± 2.75	20.7 ± 1.17	0.41 ± 0.07	42.9 ± 1.84	6.33 ± 0.50	36.8 ± 4.53	24.1 ± 0.55
BME 25	422.7 ± 18.1^a^	57.7 ± 2.05^a^	24.6 ± 1.11^a^	0.72 ± 0.02^b^	151.6 ± 0.68^c^	3.24 ± 0.26^c^	15.3 ± 0.87^c^	19.3 ± 0.29^c^
VTE 200	461.5 ± 11.5^b^	59.1 ± 3.40^a^	24.5 ± 0.88^a^	0.79 ± 0.04^c^	210.4 ± 0.84^c^	1.78 ± 0.15^c^	15.2 ± 1.53^b^	14.6 ± 0.37^c^

Values are mean ± SEM of 6 rats in each group. ^a^ *P* < 0.05, ^b^ *P* < 0.01, and ^c^ *P* < 0.001 compared to respective control group (statistical analysis was done by one-way analysis of variance followed by Dunnett's test for multiple comparisons).

**Table 3:** Effect of BME and VTE on dry granulation tissue, protein, hydroxyproline, hexosamine, and hexuronic acid content.

Oral treatment (mg/kg, od × 10 day)	Dry tissue mg/100 g bw	Protein mg/g dry tissue	Connective tissue parameters		
			Hydroxyproline *μ*g/mg protein	Hexosamine *μ*g/mg protein	Hexuronic Acid *μ*g/mg protein
Control 0.5% CMC	71.2 ± 3.80	244.1 ± 14.4	145.7 ± 12.6	86.6 ± 7.02	20.8 ± 3.94
BME 25	85.0 ± 3.70^a^	288.4 ± 10.3^a^	204.2 ± 9.44^b^	122.1 ± 7.70^b^	52.6 ± 2.90^c^
VTE 200	92.2 ± 2.0^c^	295.5 ± 17.0^a^	191.1 ± 11.2^b^	126.2 ± 8.97^b^	46.0 ± 2.86^c^

values are mean ± SEM of 6 rats in each group. ^a^ *P* < 0.05, ^b^ *P* < 0.01, and ^c^ *P* < 0.001 compared to respective control group (statistical analysis was done by one-way analysis of variance followed by Dunnett's test for multiple comparisons).
